# Dual *Arnica montana* and *Ruscus aculeatus* Hyaluronic Acid-Modified Nanostructured Lipid Carriers for Accelerated Wound Healing Effect

**DOI:** 10.3390/antiox15050594

**Published:** 2026-05-08

**Authors:** Ioana Lăcătusu, Robert Tincu, Mihaela Bacalum, Diana Lavinia Stan, Ovidiu Cristian Oprea, Mihaela Neagu, Justinian Andrei Tomescu, Nicoleta Badea

**Affiliations:** 1 Faculty of Chemical Engineering and Biotechnologies, National University of Science and Technology Politehnica Bucharest, Polizu No. 1, 011061 Bucharest, Romania; ioana.lacatusu@upb.ro (I.L.); robert_andrei.tincu@upb.ro (R.T.); ovidiu.oprea@upb.ro (O.C.O.); 2 Research Centre for Environmental Protection and Ecofriendly Technologies (CPMTE), National University of Science and Technology Politehnica Bucharest, Polizu No. 1, 011061 Bucharest, Romania; 3 C.D. Nenitzescu Institute of Organic and Supramolecular Chemistry, Romanian Academy, 202B Spl. Independentei, 060023 Bucharest, Romania; 4 Department of Life and Environmental Physics, Horia Hulubei National Institute of Physics and Nuclear Engineering, Reactorului Street No. 30, 077125 Magurele, Romania; bmihaela@nipne.ro (M.B.); diana.stan@nipne.ro (D.L.S.); 5 Academy of Romanian Scientists, 3 Ilfov St., 050044 Bucharest, Romania; 6 S.C. Hofigal Export Import SA, Intrarea Serelor No. 2, 042124 Bucharest, Romania; mihaela.neagu@hofigal.eu (M.N.); andrei.tomescu@hofigal.eu (J.A.T.)

**Keywords:** wound healing, *Arnica montana*, *Ruscus aculeatus*, lipid nanocarriers, hyaluronic acid, antioxidant activity

## Abstract

Skin wound healing involves a delicate balance between proliferation and remodelling processes, with significant therapeutic challenges. The present work aimed to investigate the capacity of hybrid lipid nanocarriers carrying a complex phytochemical profile (HA-NLC-*ArnicaM* and/or *RuscusA* extracts) to counteract the destructive action of oxidative free radicals and to accelerate wound closure induced on BJ fibroblast cells. The lipid and hybrid nanocarriers have main diameters ranging from 145 nm to 180 nm, electrokinetic potential between −45 mV and −62 mV, and entrapment efficiency of plant extracts exceeding 96%. HA-NLC-plant extracts exhibit an appropriate level of biocompatibility at concentrations < 50 µg/mL. *ArnicaM* wins the antioxidant contest while *RuscusA* proved excellent for accelerating the wound closure process. NLCs and HA-NLCs entrapping *ArnicaM* manifested the highest capacity to neutralise DPPH free radicals, reaching 79.4% inhibition. BJ fibroblast cells treated with HA-NLCs closed the wound more rapidly than NLCs, with cells reaching maximum wound closure efficiency when treated with 12.5 and 100 µg/mL HA-NLC-*RuscusA,* followed by HA-NLC-*ArnicaM-RuscusA.* These results facilitate the design of remarkable hybrid lipid nanocarriers, which exploit the emergence of a pharmacological phytochemical’s synergy, and which could contribute to stimulating signalling pathways and promoting appropriate cellular regeneration, needed for wound healing.

## 1. Introduction

Wound healing is a complex biological event essential for tissue repair and regene-ration. A wound is a disruption in the skin structure, resulting from burns, mechanical and chemical injuries, or because of the presence of an underlying medical or physiological condition [1]. Wound healing involves a balance between prolonged inflammation, proliferation, and remodelling processes, which poses a significant clinical challenge [2,3]. Conventional methodologies usually face constraints such as the risk of infection and extended healing time [4]. The efficacy of phytoconstituents in the treatment of wound healing has become a frequently mentioned approach due to their anti-inflammatory, antibacterial, and antioxidant properties. Despite the efficacy of bioactive plant mixtures, the obstacles associated with low bioavailability and inadequate stability remain significant concerns [5]. Nanostructured delivery systems are considered a hopeful solution for these phytoconstituents, especially since many of them exhibit excellent biocompatibility, closely mirror the structure of the extracellular matrix, and possess the crucial ability to maintain an environment conducive to wound healing [6].

There is a diverse range of organic nanostructures, such as natural polymeric nanomaterials (polysaccharides, proteins, and peptides) that have proven their efficacy in the wound healing in various ways, including antimicrobial properties, targeted delivery of the active principle to the wound site, promotion of angiogenesis, immunomodulation, and tissue regeneration [7]. Among the different types of nanocarriers, Nanostructured Lipid Carriers (NLCs) are gaining more popularity for topical pharmacological applications because these nanocarriers can load higher payload, avoid drug leakage, improve skin hydration, and facilitate the penetration of bioactive principles deeper into the skin [8]. NLCs were first developed in 1999, being composed of solid lipids and, to a lesser extent, liquid lipids, are biocompatible and biodegradable, and are stabilised by one or more emulsifiers [9]. Owing to their imperfections in the core structure, NLCs have a less ordered structure than other lipid nanoparticles, e.g., Solid Lipid Nanoparticles (SLNs), which allows them to carry more hydrophobic actives and keep them stable during storage [10]. NLCs improve the bioavailability of the hosted actives, protect them from degradation, enable targeted delivery, and make the delivery system more efficient at lower doses. Furthermore, NLCs are not only beneficial in improving the characteristics of lipophilic actives but may also offer a promising way to simultaneously deliver hydrophilic actives [11,12]. NLCs possess the potential to deliver herbal actives and may also play a crucial role in the management of chronic wounds. For example, NLCs loaded with St. John’s wort extract have shown a strong wound healing effect and reduced oxidative stress and tissue damage, while improving enzyme levels [13]. Coupling the NLC advantages with hyaluronic acid (HA) extends their biocompatibility and makes them more precise and specific to certain conditions. HA is a natural hydrophilic glycosaminoglycan composed of N-acetyl-D-glucosamine and D-glucuronic acid units. Being a key component of the extracellular matrix HA possesses targeting action and enhances physicochemical properties, including viscoelasticity and mucoadhesiveness [14], which extends its utility in the targeting of various diseases. HA also forms a protective hydration layer that contributes to the NLC stability and prevents the premature active release in the biological environment. In addition to these desirable characteristics, HA increases the NLC hydrophilicity and reduces non-specific protein adsorption, which may prolong the systemic circulation [15]. Recent research highlights that HA-SLNs carrying different synthesis actives, e.g., gemcitabine hydrochloride, irinotecan, dexamethasone [16], or of plant origin, e.g., silybin, presents suitable nano-platforms for MDA-MB-231 tumour cells or colon cancer-targeted therapy [17], treatment for hearing loss [18], and achieved accurate medication for alcoholic liver injury [19]. These HA-SLNs reduced drug resistance, decreased tumour size, improved the retention time, regulated the antioxidant levels, and assured re-endothelialisation in vascular wound healing. Optimised targeted HA-conjugated NLCs co-loaded with atorvastatin and curcumin improved the efficacy against glioblastoma, exhibiting eight-fold greater efficacy than free drugs [20]. Peleje et al. designed HA-coated NLCs with docetaxel for high cytotoxicity in MCF-7 breast cancer cells [15]. HA-SLNs and HA-NLCs improved the in vivo pharmacokinetics, showed greater cytotoxic effects, assured reduction in liver accumulation, inhibited cell adhesion, or promoted endothelial cell proliferation. Moreover, the remarkable results obtained on phytochemicals entrapped into HA-NLCs, hesperidin [21], and curcumin [22] underline that these HA-NLCs are suitable to improve stability, cellular absorption, and the effectiveness of the natural phytochemicals and treatment outcomes for various diseases.

**Figure 1:**
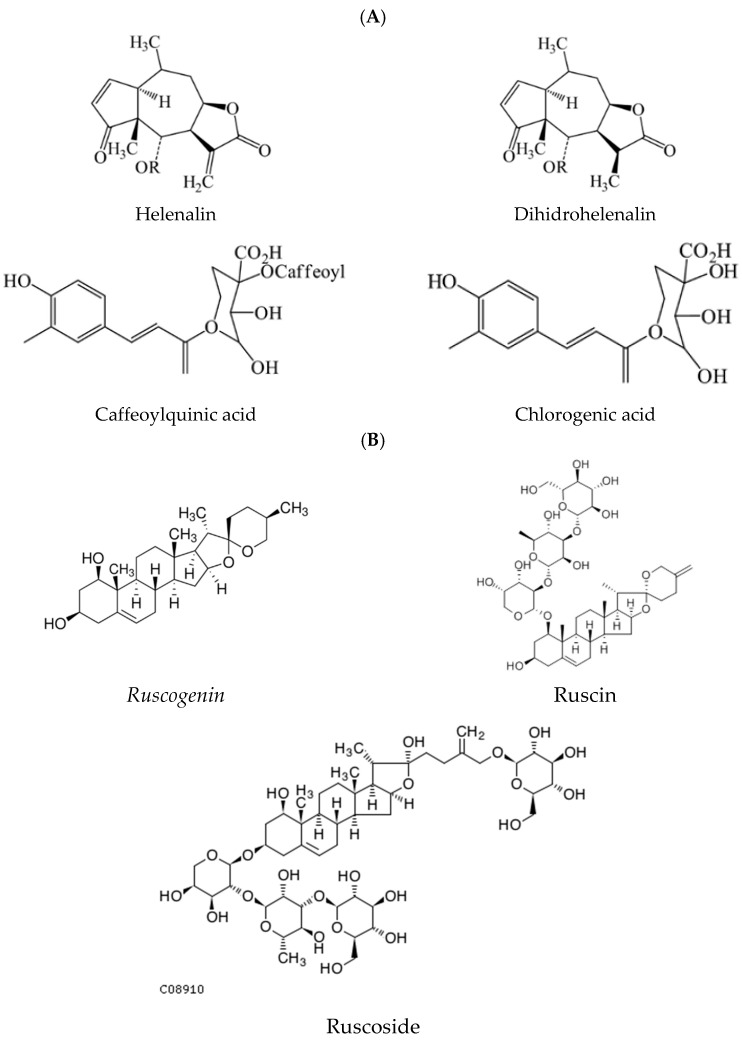
Principal phytoconstituents from *ArnicaM* (**A**) and *RuscusA* (**B**).

Given the ongoing popularity and medicinal performance of phytochemicals, the bioactive properties of *Arnica montana* (*ArnicaM*) and *Ruscus aculeatus* (*RuscusA*) extracts can be effectively harnessed through HA-NLCs, supporting their application in skin wound healing disease. The medicinal uses of *ArnicaM* extract were supported through scientific pharmacological reports, which revealed analgesic activity, circulatory stimulation, anti-arthritis action, a decrease in inflammation markers, and oxidative stress [23,24]. *ArnicaM* was used as a homeopathic remedy and is available as a topical ointment or oral formulation, being used in the treatment of trauma-associated pain and swelling [25]. The World Health Organization recognised the uses of *ArnicaM* as a topical treatment of pain and inflammation [26]. More than 150 therapeutically active compounds have been determined in *ArnicaM* extract, including polyphenolic compounds, sesquiterpene lactones (helenalin, dihydrohelenalin), flavonoid glycosides, flavonoid glucuronides (kaempferol, quercetin, and luteolin derivatives), carotenoids, diterpenes, coumarins, phenolic acids (caffeic acid and its derivatives, dicaffeoylquinic acids, ethyl caffeate), and oligosaccharides [27], Figure 1. *RuscusA* extract is nowadays recognised as a traditional herbal for therapy of minor venous circulatory disturbances and hemorrhoids [28]. The rhizome and root of *RuscusA* have been employed in folk medicine and modern phytotherapy for various dermatological and urinary ailments, haemorrhoids, and particularly in the management of disorders associated with venous insufficiency [29]. These therapeutic outcomes are attributed primarily to the presence of steroidal saponins such as ruscogenin and neoruscogenin, as well as bioactive flavonoids. *RuscusA* has revealed a complex chemical profile dominated by steroidal saponins; the characteristic constituents are ruscogenin, ruscoside, and ruscin [30]. Steroidal saponins possess a C27 carbon skeleton and contain one or more sugar residues; sugar chains are composed of α-L-rhamnose, β-D-galactose, and β-D-glucose units. Beyond saponins, *RuscusA* contains a diverse array of phenolic compounds and flavonoids: derivatives of apigenin, quercetin, and kaempferol, including apigenin-C-hexoside-C-pentoside, quercetin-O-deoxyhexoside-hexoside, caffeic acid hexoside, and kaempferol-O-deoxyhexoside-hexoside [29]. In clinical tests, it was shown that *RuscusA* decreases the capillary filtration rate and reduces the vascular permeability in healthy volunteers and patients with chronic venous disease [31]. These remarkable results have made the *RuscusA* extract available on the market in various pharmaceutical and cosmetic preparations, particularly those intended to improve venous tone, reduce swelling, and alleviate symptoms of chronic venous disorders.

Starting from *RuscusA* and *ArnicaM* therapeutic outcomes, there is a compelling need to complement the existing scientific data on these extracts with current knowledge in the field of polymer-decorated NLCs. Although several previous studies have explored HA-NLCs, they have primarily concentrated on the individual synthetic or natural active delivery, aiming at antitumour properties, management of antiobesity, or to alleviate intestinal inflammation and maintain gut homeostasis. To our knowledge, no prior study has reported the development of HA-coated NLCs loaded with *RuscusA* and *ArnicaM*, which represent a unique functional approach. This distinction is important given the superior and tuneable characteristics of HA-NLC coupled with the bioefficacy of *RuscusA* and *ArnicaM* in the treatment of skin diseases. The primary innovation of this work consists in investigating for the first time the comparative capacity of hybrid lipid nanocarriers to counteract the destructive action of oxidative free radicals and to accelerate wound closure induced on human skin-derived BJ fibroblast cells. The efficacy and potential success of HA-NLCs that entrap *ArnicaM* and/or *RuscusA* is based on the HA property to orchestrate the active phytochemicals so that they trigger intracellular signalling cascades that act simultaneously as engines of cell regeneration. The study impacts through the synergy between the structural profile of the active phytocompounds from *ArnicaM* and *RuscusA* extracts correlated with the unique structural abilities of the hybrid carbohydrate–lipidnanocarrier to stabilise, deliver, and gradually release the phytochemical actives.

## 2. Materials and Methods

### 2.1. Materials

Hyaluronic acid sodium salt (from *Streptococcus equi*), Folin–Ciocâlteu reagent, gallic acid, 2,2-diphenyl-1-picrylhydrazyl, ethanol Chromasolv^®^, phosphate buffer (PBS), and poly(ethylene glycol)-*block*-poly(propylene glycol)-*block*-poly(ethylene glycol)/Poloxamer 188 were supplied by Sigma Aldrich Chemie GmbH (Schnelldorf, Germany). Soy lecithin was purchased from Alfa Aesar, and Tween 20 (polyoxyethylenesorbitan monolaurate) was purchased from Merck (Germany). The lipids: glycerol monostearate (GMS) was purchased from Cognis GmbH (Monheim am Rhein, Germany) and mango butter from Sobio Cosmetics SRL (Bucharest, Romania). The milk thistle was purchased from Elemental (Oradea, Romania) with the following fatty acids composition: 54.5% linoleic acid (ω-6), 39.67% oleic acid (ω-9), 8.67% palmitic acid, 6.27% methyl stearate. The argan oil was purchased from SC Herbavit SRL (Oradea, Romania) with the following fatty acids composition: 30.8% linoleic acid (ω-6), 49.2% oleic acid (ω-9), 13.93% palmitic acid, 5.59% methyl stearate. The vegetal extracts *Arnica montana* and *Ruscus aculeatus L.* were procured from S.C. Hofigal Export Import SA (Bucharest, Romania). The two extracts with a phytochemical profile rich in polyphenols, flavonoids, glycosides and glucuronides, phenolic acids, as well as terpene lactones and steroidal saponins were isolated from the flowers and roots of *ArnicaM* and *RuscusA* by the ultrasound-assisted extraction method (1 h, 40 °C, 80 kHz). *ArnicaM* extract has a polyphenol content of 57 mg GAE/g lyophilised and a total flavone content of 220.76 mg/g lyophilised extract (expressed in quercetin equivalents). The determined amounts of polyphenols from *RuscusA* extract were 29.03 mg GAE/g lyophilised and with a total flavonoid content of 18 mg/g lyophilised extract.

### 2.2. Nanostructured Lipid Carrier and HA-Modified NLC Preparation

Free NLCs and phytochemical extract-loaded NLCs were prepared using the melt emulsification method coupled with high-pressure homogenisation, as previously described by the authors [32,33]. Briefly, the lipid phase, composed of glycerol monostearate, mango butter, milk thistle, and argan oil, and the aqueous phase, comprising the surfactant mix (Tween 20, Poloxamer 188, and soy lecithin) with phytochemical extracts, were combined at 70 °C and the obtained pre-emulsion was kept under constant stirring at the same temperature for 15 min. Consequently, each pre-emulsion was processed by HSH (High Shear Homogenizer PRO250, Oxford, CT, USA) at 12,000 rpm for 1 min and HPH (APV 2000 Lab Homogenizer, Holzwickede, Germany) for six homogenisation cycles at 500 bars, for 196 seconds.

For the preparation of HA surface-decorated NLCs, 30 mL of HA aqueous solution (2 mg/mL) was added dropwise under vigorous stirring to 60 mL of NLC dispersion at 37 °C. The resulting nanodispersions were cooled to room temperature, stored overnight at −25 °C, and subsequently freeze-dried by lyophilisation (−55 °C, 0.05 mbar, 54 h) using a Martin Christ Alpha 1–2 LD freeze dryer (Osterode am Harz, Germany).

### 2.3. Size and Physical Stability Analysis

Dynamic light scattering (DLS) was used to measure the mean particle diameter (Z_ave_) and polydispersity index (PdI) of synthesised NLC systems using a Zetasizer ZS 90 (Malvern Instruments Inc., Worcestershire, UK), equipped with a solid-state laser (670 nm) at a scattering angle of 90°. DLS samples were obtained following the dilution of the NLC aqueous dispersion to attain a suitable scattering intensity.

Physical stability of the NLC dispersions was assessed by measuring the surface charges using the Zetasizer ZS 90 (Malvern Instruments Inc., Worcestershire, UK) in a capillary cell. Following the analysis, the obtained electrophoretic mobility was converted to zeta potential using the Helmholtz–Smoluchowski equation (Equation (1)):

(1) $$\xi =EM\frac{4\pi \eta }{\varepsilon }$$

where ξ represents zeta potential, EM represents electrophoretic mobility, η represents viscosity of the dispersion medium, and ε represents the dielectric constant.

### 2.4. Phytochemical Extracts Entrapment Efficiency

The entrapment efficiency of *Arnica montana* and *Ruscus aculeatus* extracts was assessed by determining the total content of polyphenols extracted in water from a known amount of lyophilised NLCs and HA-NLCs. The total polyphenol content was quantified using the Folin–Ciocâlteu assay and expressed as gallic acid equivalents (GAEs) [34]. In short, 0.15 g of lyophilised NLC was suspended in 1 mL of water, gently shaken, and then subjected to 5 min of centrifugation at 15,000 rpm. An amount of 0.5 mL of supernatant was sampled, mixed with 2 mL of Folin–Ciocâlteu reagent 10% (*v*/*v*) and 2.5 mL of Na_2_CO_3_ 7.5%, and incubated for 1 h in the dark at room temperature. The absorbance was measured at λ = 765 nm in triplicate (UV–Vis spectrophotometer V670 Jasco, Tokyo, Japan) and the concentration of polyphenols was calculated against a gallic acid calibration curve (R^2^ = 0.9919, range 0–100 mg/mL). Entrapment efficiency of phytochemical extracts (EE) was computed using Equation (2):

(2) $$EE\left(\%\right)=\frac{C_{NLC}}{C_{extract}}\cdot 100$$

where $C_{NLC}$ represents polyphenol content entrapped in NLC and $C_{extract}$ represents polyphenol content of *Arnica montana* or *Ruscus aculeatus*.

### 2.5. In Vitro Antioxidant Activity

In vitro antioxidant activity of the free and loaded NLC systems was determined using the 2,2-diphenyl-1-picrylhydrazyl (DPPH) assay. Practically, 3 mL of DPPH ethanolic solution (0.04 mg/mL) was mixed with 2 mL of NLC solution (2 mg/mL) and incubated for 30 min in the dark. Consequently, the sample was analysed by UV–Vis spectroscopy (UV–Vis spectrophotometer V670 Jasco, Tokyo, Japan) at λ = 517 nm, and the scavenging effect was calculated using Equation (3):

(3) $$\%Inhibition\;\mathrm{DPPH}=\frac{A_{c}-A_{s}}{A_{c}}\cdot 100$$

where $A_{c}$ represents absorbance of the control (sample prepared in the same manner, but replacing the NLC solution with ethanol) and $A_{s}$ represents absorbance of the NLC sample. Each sample was analysed in triplicate.

The IC_50_ values (mg/mL), corresponding to the concentration required to inhibit 50% of DPPH radicals, were calculated from the regression of the inhibition percentage versus sample concentration (0.5 ÷ 2 mg/mL).

### 2.6. Differential Scanning Calorimetry

Differential scanning calorimetry (Netzsch DSC 204 F1 Phoenix, Selb, Germany) was employed to investigate the thermal properties of the freeze-dried NLCs. Samples (approximately 20 mg; free and/or loaded NLCs) were placed in sealed aluminium pans and subjected to a temperature program from 25 to 100 °C at a heating rate of 10 °C/min under a nitrogen atmosphere (40 mL/min). An empty aluminium pan served as the reference.

### 2.7. In Vitro Release of ArnicaM and RuscusA

Studies regarding the in vitro release of polyphenols from both the NLC- and HA-NLC-type of formulations were conducted using a Franz diffusion cell (Hanson Research Corporation, Chatsworth, CA, USA). The typical experiment is carried out as follows: the receptor chamber is filled with the medium (ethanol: water = 1:1 *v*/*v*), while 200 µL of NLC dispersion is placed in the donor chamber on a Strat-M^TM^ Membrane, Transdermal Diffusion Test Model (Millipore, Germany). The receptor medium is maintained at 37 °C, under constant stirring at 400 rpm, and at specific 1 h time intervals, 0.5 mL of sample is withdrawn from the receptor chamber for quantitative analysis (see Section 2.4) and simultaneously replaced with an equal volume of fresh receptor medium. The experiment was conducted for 7 h.

The kinetics of polyphenol release were determined through the application of five mathematical model equations: zero order, first order, Higuchi, Hixson–Crowell, and Peppas–Korsmeyer [35,36]. The optimal model for the release kinetics was selected based on the highest R^2^ value.

### 2.8. BJ Skin Fibroblast Cells

Human skin fibroblast cells (BJ, ATCC^®^, CRL-2522™, Manassas, VA, USA) were cultured in DMEM (Dulbecco’s modified Eagle medium) supplemented with 10% fetal bovine serum (Gibco) and 100 U/mL penicillin with 100 μg/mL streptomycin (Gibco), in a humidified atmosphere of 95% air/5% CO_2_ at 37 °C. Depending on the experiments, various cell densities were used, as mentioned below. All cell cultivation media and reagents were purchased from Gibco (New York, NY, USA).

### 2.9. Cell Viability Assay

The cell viability assay was performed in a 96-well plate with 7000 cells seeded into each well. After 24 h, cells were treated for an additional 24 h or 48 h with various concentrations of the compounds (0, 6.25, 12.5, 25, 50, 100 µg/mL). Following the desired time of treatment, the medium was replaced with a 1 mg/mL MTT solution (Serva, Heidelberg, Germany) and further incubated for 3–4 h at 37 °C. After this time, the medium was removed, and the purple formazan crystals formed by the metabolically active cells were dissolved using 100 µL of DMSO. Finally, the solution absorbance was measured at 570 nm using a Mithras LB 940 plate reader (Berthold Technologies, Bad Wildbad, Germany) and cell viability was calculated relative to untreated controls. All experiments were performed in triplicate and repeated at least three times.

### 2.10. Release of the Cellular Damage Marker, Lactate Dehydrogenase (LDH)

LDH release was measured using the Invitrogen™ CyQUANT™ LDH Cytotoxicity (ThermoFisher Scientific, Waltham, MA, USA). Assay, and the same plating as before was used. After the desired treatment time, 50 µL of the medium in each well was transferred to a 96-well flat-bottom plate and mixed with an equal volume of reaction medium. The plate was incubated for 30 min, protected from the light, at room temperature. After, 50 µL of Stop Solution was added to each well and mixed by gentle tapping. Finally, the absorbance measured at λ = 490 nm and LDH levels were calculated compared to the control condition. All experiments were performed in triplicate and repeated at least three times.

### 2.11. Morphological Evaluation by Fluorescence Microscopy

The morphological changes were investigated by staining the actin filaments with Phalloidin-FITC (Sigma-Aldrich, Saint Louis, MO, USA) and imaging them using an Olympus BX-51 epifluorescence microscope (Olympus, Düsseldorf, Germany), equipped with a 40× objective. For this, the cells were grown on a cover glass in the presence of the two concentrations of the samples (12.5 and 100 µg/mL) for 24 h. Then, the cells were washed with phosphate-buffered saline (PBS) three times, fixed with 4% formaldehyde, rewashed three times with PBS, permeabilised with 0.1% Triton X-100 in PBS, and washed again with PBS three times. Finally, the fluorescent dye was added to the cells and left in the dark, at room temperature, for 1.5 h. In the last step, the cells were washed with PBS three times and fixed with FluorSaveTM (Merck, Darmstadt, Germany). The images were further pseudo-coloured using the ImageJ software (version 1.53a, Madison, WI, USA). Further, the images were analysed using a MATLAB R2020a routine reported previously to evaluate the cytoskeleton changes [37].

### 2.12. Wound Healing Assay

Cell migration ability of BJ cells treated for 24 h with two concentrations (12.5 and 100 µg/mL) was assessed by the scratch assay. Cells were first grown in a confluent mo-nolayer using an Ibidi Culture-Insert 2 Well, creating a wound with a 500 µm ± 100 µm width. Following the removal of the inserts, the cells were treated with the NLCs and HA-NLCs systems at the desired concentrations and imaged at baseline several times (4, 24, and 30 h) after exposure, using an inverted microscope (Olympus CX23 Binocular Microscope, Düsseldorf, Germany) equipped with a 10× objective. Each condition was tested in triplicate, and the experiment was repeated at least twice on different cell passages. The wound width and the percentage of wound closure were determined as described previously [38].

## 3. Results

### 3.1. Size Parameters and Physical Stability Assignment of NLCs and Carbohydrate Modified-NLCs Co-Loaded with ArnicaM and/or RuscusA

**Figure 2:**
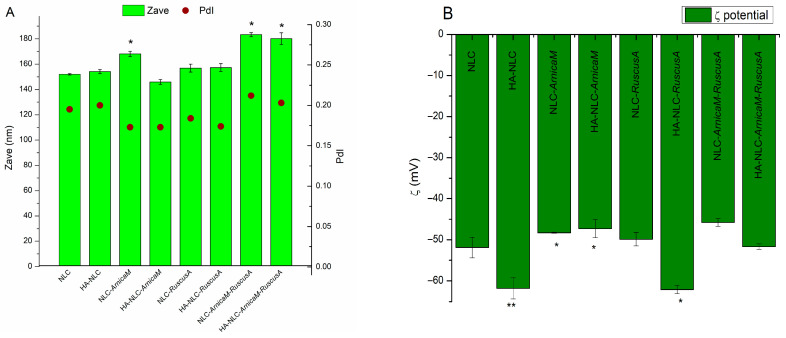
DLS (**A**) and zeta potential analysis (**B**) of the synthesised NLC systems. * *p* < 0.05; ** *p* < 0.005; data are expressed as mean ± SD, *n* = 3 NLCs vs. other groups.

DLS and zeta potential measurements reveal the formation of NLC dispersions with physical characteristics suitable for dermal applications. All formulations showed dimensions less than 200 nm (even less than 150 nm in the case of HA-NLC-*ArnicaM*), with PDI values around 0.2, thus demonstrating the affinity and robust design of the matrix lipid for encapsulating complex vegetal extracts (Figure 2A). Electrokinetic potential has negatived enough values to ensure physical stability by electrostatic repulsions. In addition to these general observations, some insightful information can be obtained; for instance, by coating the free NLC formulation with HA, a considerable change in the zeta potential (−51.9 ± 2.49 mV for NLCs vs. −61.8 ± 2.51 mV for HA-NLCs) has been produced (Figure 2B). As other studies are reporting [39,40], this result is due to the carboxylate groups from the HA, hence confirming the successful attachment of the polysaccharide to the surface of NLCs. Loading the NLC formulation with *ArnicaM* extract leads to a slight increase in particle size, probably due to accommodation of the most polyphenolic compounds in the surfactant layer, a hypothesis confirmed by the characteristics of HA-NLC-*ArnicaM* (smaller average diameter and fewer negative values of zeta potential). Localisation of these compounds in the surfactant layer facilitates the interaction with HA through non-covalent interactions, promoting a tighter packing of the “layers” and partially shielding the negative charges [41]. On the other hand, the changes in characteristics of NLC-*RuscusA* and HA-NLC-*RuscusA* are not particularly significant, possibly due to the amphiphilic nature of Rucosgenin, a major steroidal saponin component of *RuscusA* extract [29]. Incorporation of both extracts in equal quantities shows a slight increase in the average particle size, although remaining under 200 nm, proving the capacity of this matrix to accommodate high amounts of active principles.

### 3.2. Structural Modifications in Nanocarriers and Hybrid Carbohydrate–Lipid Nanocarriers, by Scanning Calorimetry and FT-IR Analysis

**Figure 3:**
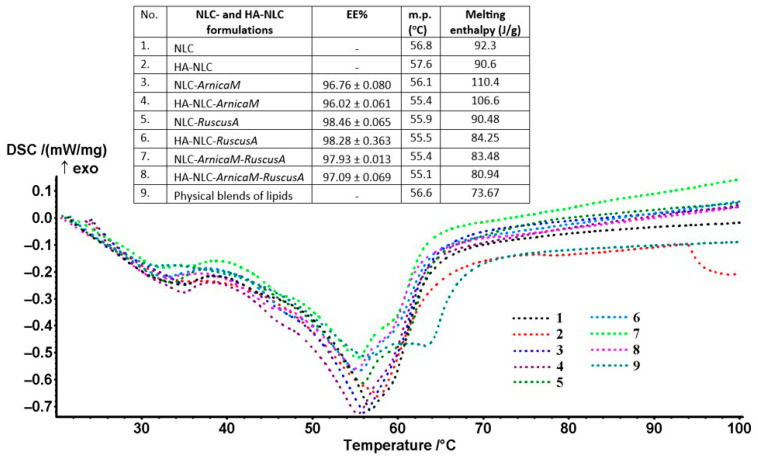
Thermal structural behaviour of NLC- and HA-NLC-plant extracts and entrapment efficiency of *ArnicaM* and *RuscusA* extract.

The results obtained from differential scanning calorimetry (DSC) provide detailed insights into the thermal modifications and amorphous state of the free and phytoche-micals-loaded NLCs and of those of NLCs covered with polymeric HA. The phytochemical mixtures of *ArnicaM* and *RuscusA* were effectively stabilised in the nanocarrier systems due to the arrangement’s sufficient and appropriate host capacity of NLCs and HA-coated-NLCs. Figure 3 illustrates the DSC curves for the NLC and HA-NLC formulations containing individual phytochemical mixtures, as well as those mixed, *ArnicaM* and *RuscusA.* The DSC thermograms revealed the existence of pronounced endothermic peaks, with maxima located in the range of 55.1 ÷ 57.6 °C, accompanied by obvious modifications of the melting enthalpies (Figure 3). Although the peak shapes are somewhat similar between the NLC categories, changes in melting point and enthalpy are noticeable, indicating significant interactions between the developed NLCs and HA-decorated NLCs. DSC behaviour confirms the solid and amorphous nature of NLCs and shows perturbations of the complex lipid core; NLCs and HA-NLCs melted in a broad temperature range because of the presence of structural differences of the solid and herbal fats, in a ratio of 1:0.66:0.55 = MSG:argan oil:mango butter. This behaviour is characteristic of lipids with an internal structure that is not so well organised.

The identification of distinct thermal characteristics of NLC- and HA-NLC-*ArnicaM* and/or *RuscusA* compared to free NLCs and HA-NLCs supports the efficient integration of phytochemicals inside these lipid nanocarriers. The thermal profile modification, i.e., variation of the melting points and of the enthalpy recorded for the free HA-NLCs *versus* the HA-NLCs loaded with *ArnicaM* and/or *RuscusA* (aprox. 2 °C difference between HA-NLCs and HA-NLC-herbal extracts), demonstrates that a preferential distribution of phytochemicals has successfully occurred. For example, the endo effect from HA-NLCs (without herbal extract) recorded at 57.6 °C has decreased up to 55.1, 55.4, and 55.5 °C in the HA-NLCs loaded with *ArnicaM* and/or *RuscusA* extracts. The additional components, e.g., phytochemicals and HA to the surfactant-stabilised lipid matrix, induce a decrease in the melting point, a phenomenon known in thermodynamics as “cryoscopic depression” [42]. Even if these phytochemicals are not fully entrapped inside the lipid core, their presence at the interface modifies the surface tension and the free energy of the entire nanocarriers system. The DSC data obtained were consistent with reports from other related studies [43], suggesting well-defined melting transitions, consistent with the nature of the complex lipid blend, which requires a significant amount of energy to melt. The decreasing melting enthalpy following the coating of NLCs with HA is a well-documented phenomenon in the literature. This modification is mainly due to the interactions at the molecular level between the HA polymer and the NLC surface [44]. Hyaluronic acid is a hydrophilic polymer that partially penetrates between the polar ends of amphiphilic lipids, surfactants, and phytochemicals, thus disrupting the organisation and recrystallisation process of the lipid matrix. This interaction forces a rearrangement of the molecules, leading to a less ordered structure of the lipid core, and, respectively, an amplification of imperfections in the lipid network. A more amorphous matrix, with a less organised structure, requires less thermal energy to pass from the solid to the liquid phase. Also, this “enclosure” of the NLC with the HA polymer can exert a certain pressure or forces that slightly destabilise the fatty acid chains packing from the MSG/mango butter and argan oil inside the nanocarriers [45].

The higher ΔH values of NLC-herbal extracts compared to those of the HA-decorated NLC-herbal extracts suggest a more cohesive structure in the conventional NLC (as compared with the HA-NLC), which requires more energy to melt. On the other hand, the lower enthalpy detected in the case of HA-NLC-*ArnicaM* and/or *RuscusA* extracts compared to NLC-*ArnicaM* and/or *RuscusA* (e.g., 110.4 J/g and 106.6 J/g for NLC- and HA-NLC-*ArnicaM*; 83.48 J/g and 80.94 J/g for NLC- and HA-NLC-*ArnicaM-RuscusA*) suggests that herbal extracts implicitly modify the organisation of the lipid core–surfactant shell, reducing the energy required for the phase transition. Both HA and *ArnicaM* and *RuscusA* form hydrogen bonds and electrostatic interactions with the components of the nanocarrier system. A lower enthalpy indicates specific interactions between HA, phytochemicals, and surfactants that reduce the melting energy. These interactions “consume” part of the energy of the system and prevent the lipid molecules from arranging themselves in a perfect crystalline structure. As a result, the addition of HA to NLCs demonstrates favourable characteristics for the plant extracts delivery, and the lower melting enthalpy can improve the robustness of NLCs during storage and under biological conditions. In addition, a lower enthalpy (and implicitly a less organised lipid matrix, with reduced crystallinity) is often an advantage in nanocarrier formulations, since a less “tight” matrix allows a higher loading capacity with active principles and prevents their expulsion during storage (a desirable feature for controlled release systems).

**Figure 4:**
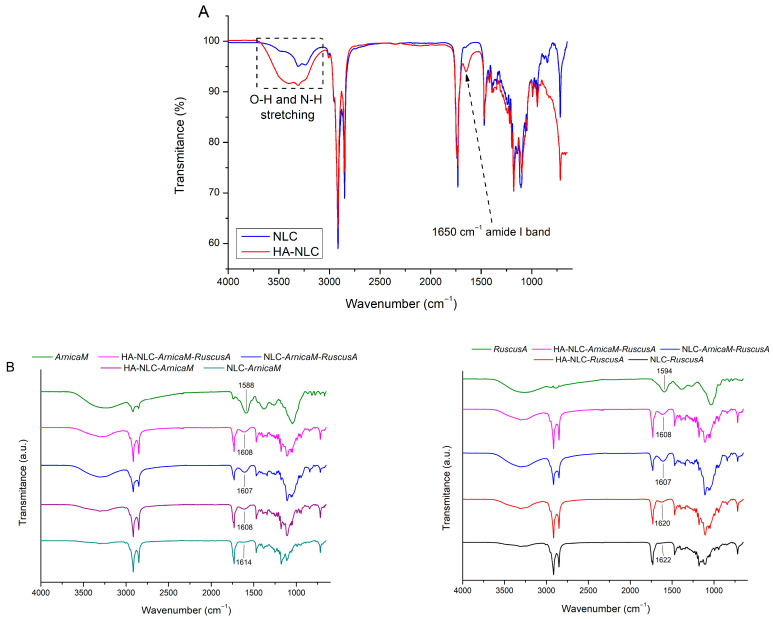
Comparative FTIR spectra of NLCs and HA-NLCs (**A**) and of phytochemicals extract entrapped into NLCs and HA-NLCs (**B**).

The ATR-FTIR spectra of NLC- and HA-NLC-plant extracts (Figure 4) showed the absorption bands of various types of chemical bonds found in the surfactants, lipids, *ArnicaM* and *RuscusA* extracts, along with hyaluronic acid (Figure 4). Both phenolic acids (such as caffeic, ferulic, and chlorogenic acids derived from *ArnicaM* and *RuscusA*) and flavonoids (including quercetin, kaempferol, luteolin, apigenin, etc.) produce specific bands in the FT-IR spectrum that are characteristic of polyphenolics. For instance, free -OH groups or those linked through hydrogen bonds are identified in the 3400–3200 cm^−1^ range as a broad, intense band. Aliphatic C–H bonds (originating from surfactants, glycosidic residues, etc.) were observed in the 2930–2850 cm^−1^ region [46]. A prominent band observed at 1750–1650 cm^−1^ is linked to the carbonyl group found in phenolic acids, or the γ-pyrone heterocycle from flavonoids. Several vibrations within the 1610–1450 cm^−1^ range could be assigned to the aromatic structure of phenols and flavonoids. The bending vibrations of the C-H bonds in the aromatic ring, which vary according to the degree of ring substitution, occur between 1000–650 cm^−1^. Generally, the specific vibrations of C-O and C-C bonds in alcohols, phenols, ethers, and flavonoid glycosides are overlapped in the range of 1320–1000 cm^−1^ [47]. The stretching of phenolic C-O bonds is noted at ~1260 cm^−1^.

A comparative study of the spectra from free NLCs and HA-decorated NLCs highlighted the presence of a new band at 1650 cm^−1^, attributed to the amide *I* stretching vibration of the N-acetyl groups in the HA backbone [48]. Furthermore, there was a simultaneous enhancement and broadening of the O-H and N-H vibrational modes in the range of 3050–3700 cm^−1^, which is consistent with the hydrogen bonding interactions between the HA molecule and NLC [49]. This effectively demonstrates the successful coating of lipid nanocarriers with HA. The FTIR spectra of HA-NLCs incorporating *ArnicaM* and/or *RuscusA* reveal slight differences in the characteristic band corresponding to the aromatic C=C stretching vibration in the polyphenol structure, e.g., 1607 and 1614 cm^−1^ for NLC-*ArnicaM,* 1607 and 1622 cm^−1^ for NLC-*Ruscus*. The slight change in the wavenumber suggests physical interactions between the phytochemicals and the functional groups from lipids and surfactants [24,50].

### 3.3. Entrapment Efficacy of ArnicaM and RuscusA into Lipid Nanocarriers and Hybrid Polysaccharide–Lipid Nanocarriers

Entrapment efficacy (EE%) is a critical parameter for assessing the nanocarriers’ ability to effectively carry and release the desired drugs. The entrapment values determined for *ArnicaM* and *RuscusA* were more than 96%, regardless of the type of lipid nanocarrier, classical NLC or HA-coated NLC. These remarkable values are justified by a structural synergy between lipid matrix, herbal extracts, and the peripheral surfactants layer of NLCs, favouring the molecular packing of vegetable extracts in both regions, hydrophilic and lipophilic. Phosphatidylcholine acts as an anchor on the polyhydroxylated phytochemicals of *ArnicaM* and *RuscusA.* Being an amphiphilic surfactant, with a high affinity for both the lipophilic and hydrophilic phases, it can establish hydrogen bonds or electrostatic interactions (through phosphate and choline groups) with the hydroxyl groups of sesquiterpene lactones, steroidal saponins, but also with flavonoids and phenolic acids from *ArnicaM* and *RuscusA,* respectively. Moreover, the steroidal saponins from *RuscusA* containing one or more sugar residues composed of α-L-rhamnose, β-D-galactose, and β-D-glucose units assured significant emulsifier properties [51]. Alkylsorbitans are also used to stabilise the lipid core, creating a rigidity effect, inserting themselves between phosphatidylcholine molecules, thus favouring a denser and harder shell, difficult to cross. In addition, the presence of argan oil in the NLC’s core creates multiple solubilisation “compartments”, allowing a massive loading of plant actives, without saturating the system. The creation of an imperfect lipid core that is efficiently “sealed” by alkylsorbitans confers adequate entrapping, preventing the outside diffusion of active phytoconstituents. The increased entrapment efficiency is facilitated by the chemical nature of phytoconstituents from *ArnicaM* and *RuscusA.* Both helenalin and ruscogenins have a partition coefficient favourable to the lipid phase, while polyphenolic acids and flavonoid glycosides and glucuronides prefer the hydrophilic shell of emulsifiers [52]. We have not found in the related literature the encapsulation of these extracts in NLC nanodelivery systems, but other studies have reported EE% values ranging from 96% to 98.5% for NLCs designed for carrying natural/hydrophilic actives [53]. This underlines that the determined EE values in the present study are within or even above average, suggesting that the developed NLCs and HA-NLCs exhibit remarkable entrapment ability even for complex mixtures of phytochemicals.

### 3.4. Assignment of the In Vitro Antioxidant Capacity of NLCs and HA-NLCs

**Figure 5:**
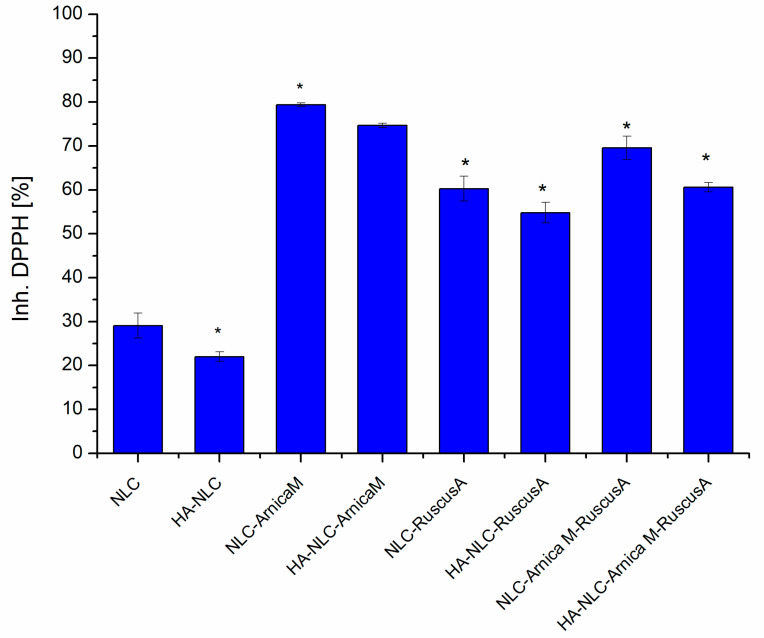
Scavenger activity of lipid nanocarriers against DPPH radicals. * *p* < 0.05; data are expressed as mean ± SD, *n* = 3 NLCs vs. other groups.

The free radical scavenging activity for NLC- and HA-decorated NLC-entrapping *ArnicaM* and/or *RuscusA* extracts was comparatively analysed using the DPPH assay (Figure 5). This evaluation aimed to illustrate the ability of the developed nanocarrier systems to capture DPPH free radicals and prevent lipid peroxidation, thus diminishing the oxidative stress-induced lesions. For all the developed NLCs loaded with *ArnicaM/RuscusA* extracts, an effective DPPH radical scavenging capacity was detected, which in some cases reached almost 80% DPPH inhibition. An increased DPPH radical scavenging capacity was recorded for the NLC and HA-NLC formulations containing *ArnicaM* (e.g., 79.38 ± 0.25%, 74.69 ± 0.50%). The nanostructured formulations with *RuscusA* showed moderate antioxidant activity (60.28 ± 2.82%), a result that is consistent with its low polyphenol content (29.03 mg GAE/g lyophilised) and flavones concentration (18 mg quercetin equivalents/g lyophilised). The superior behaviour of NLC-*ArnicaM* compared to NLC-*RuscusA* is mainly due to the distinct phytochemical structural profile of the two phytochemical extracts.

Some fundamental aspects that justify these differentiations are the following: *i. Structural profile of phytochemicals. ArnicaM* is rich in phenolic acids (chlorogenic acid, caffeic acid), flavonoids (luteolin, quercetin), and terpene lactones (helenalin, dihydrohelenalin, and their esters) [54]. These polyphenolic compounds with electron-rich substituents are excellent hydrogen atom donors and can react with DPPH radicals through a very fast electron transfer, leading to efficient DPPH radical scavenging [55]. Although effective for venous circulatory functions, the major steroidal saponins (ruscoside, ruscogenin) from *RuscusA* do not possess the same density of active phenolic hydroxyl groups as the flavonoids from *ArnicaM.* Luis et al. also indicated the limited antioxidant potential of *RuscusA* extract, which correlates with a low level of polyphenols (32.9 mg GA/g dry matter) [56]. *ii. Synergistic effect of terpene lactones in conjunction with flavonoids.* Another major differentiator for *ArnicaM* is the presence of terpene lactones (especially helenalin and its esters). Terpene lactones can interact synergistically with flavonoids [57]. This interaction stabilises the lipid phase of NLCs and enhances electron transfer, providing a higher reaction rate in neutralizing the DPPH radical compared to *RuscusA* extract. *iii. Hydrophobicity* vs. *hydrophilicity* vs. *accessibility.* The antioxidant phytocompounds in *ArnicaM* (especially methoxylated flavonoids) tend to have a more balanced affinity for the lipid matrix. This strategic location makes the active groups more accessible to the DPPH radical in the reaction medium. In the case of *RuscusA,* saponins tend to be bulkier and may reduce direct collision with the oxidant DPPH.

Regarding the dual nanocarriers, NLC- and HA-NLC-*ArnicaM-RuscusA* showed moderately increased inhibition of DPPH radicals, e.g., 69.56 ± 2.68% and 60.6 ± 2.68%, respectively. This behaviour can be explained by the presence of multiple phytomolecules, which can interfere with each other, limiting access to DPPH radicals [55]. Polyphenols will have less access to the DPPH radical site, so the electron transfer is slowed down. The result is the inhibition of the hydrogen transfer reaction necessary for the formation of the complex stabilised by conjugation (between the double bonds and the non-participating electron pair from the nitrogen or oxygen atom) [58,59].

Notable differences were also reported between NLCs vs. HA-coated NLCs. For instance, decreases of approx. 5.8% in DPPH scavenging capacity were recorded in the case of HA-decorated NLC-*ArnicaM* and of 12.9% in the case of HA-NLC-*ArnicaM-RuscusA* vs. NLC-*ArnicaM-RuscusA*. Although hyaluronic acid is a valuable ingredient for stability, improved adhesion to biological tissues, hydration, and biocompatibility, its presence on the NLC surface may act as a physicochemical barrier that “screens” the antioxidant potential of *ArnicaM* and *RuscusA* extracts. Several mechanisms that explain the decrease in antioxidant activity of HA-NLCs can be associated with the following: ***a.*** *Creation of a steric barrier.* The branched and dense polymeric layer of HA attached to the NLC surface leads to a physical barrier that limits the diffusion of the DPPH free radical to the antioxidants. The HA slows down the reaction kinetics, resulting in a decrease in DPPH inhibition [60]. ***b.*** *Sequestration of phytochemical antioxidants*. There is a high probability that polyphenols from *ArnicaM* and saponins from *RuscusA* form intermolecular hydrogen bonds with hydrophilic, carboxyl, and hydroxyl groups of HA [61]. By this “anchoring”, the active groups of the antioxidants become partially blocked in the polymer network, thus reducing the number of active centres available for neutralisation of DPPH radicals. ***c.*** *Modification of surface potential and viscosity*. Coating with HA modifies the electrokinetic properties of the nanocarriers. The HA layer increases viscosity, which reduces molecular mobility [62]. Since the DPPH reaction is rate-dependent, the reduction of molecular mobility of reactive species leads to a lower capture efficiency of DPPH radicals. The antioxidant activity exhibited by NLCs without plant extracts (29.09 ± 3.83%) can be attributed to the presence of fatty acids (ω-6 linoleic acid and ω-9 oleic acid) present in argan oil, milk thistle oil, and mango butter used for the synthesis of the lipid matrix of NLCs [63,64].

The IC_50_ values obtained for the two categories of nanocarrier systems (Table 1) reflect the antioxidant superiority of NLC- and HA-NLC-*ArnicaM* over NLC systems with *RuscusA* or dual ones. *ArnicaM* wins the antioxidant competition based on the appropriate polyphenolic structures, while *RuscusA* (with high saponin content) is excellent for venous tone but much weaker in direct neutralisation of free radicals by the DPPH method.

### 3.5. Assignment of the In Vitro Phytochemicals Release from NLCs and HA-NLCs

**Figure 6:**
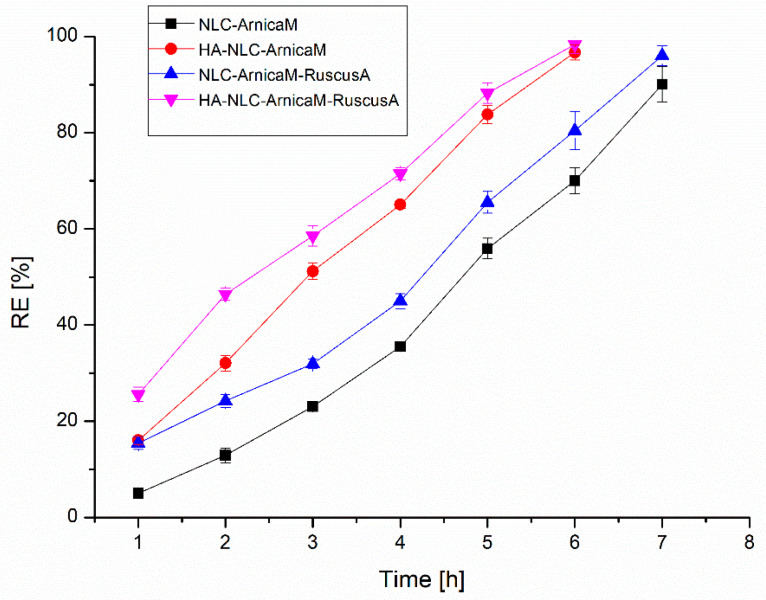
In vitro release from decorated and undecorated NLCs loaded with vegetal extract.

Evaluation of phytochemicals release from nanocarriers is essential to understand the in vivo performance of the developed NLCs and HA-decorated NLCs. In vitro release studies were performed using Franz cells on selected nanocarriers, and the amount of polyphenols released from the NLCs and hybrid HA-NLCs loaded with extracts was determined by quantitative Folin–Ciocâlteu assay. Comparative analysis of the release profiles of NLC-*ArnicaM* and NLC-*ArnicaM-RuscusA* (Figure 6) reveals a significant difference, mainly influenced by the type and amount of phytochemical mixture entrapped in the nanocarrier. In the first hour, NLC-*ArnicaM* releases 4.99 ± 0.34% of polyphenols, in contrast to the 15.46 ± 1.32% released by the NLC-*ArnicaM-RuscusA.* This approx. three-fold increase in the release percentage can be attributed to the amount of encapsulated extract, which resulted in the oversaturation of the available spaces in the NLC to accommodate both types of phytochemical blends. Polyphenols that were not captured in the internal surfactant network or that formed very weak interactions were easily taken up by the acceptor environment. After 7 h, the release was 90.02 ± 2.7% for NLC-*ArnicaM* and 96.02 ± 2.1% for NLC-*ArnicaM-RuscusA*.

The decoration of NLCs with hyaluronic acid resulted in a notable increase in the rate of polyphenol release. Consequently, for *ArnicaM*, the NLC that was decorated achieved a release percentage of 96.68% after only 6 h, compared to 90.02% for the conventional NLC measured after 7 h. A similar trend was observed in the NLC-*ArnicaM*-*RuscusA*, where the variant coated with HA recorded a release efficiency of 98.36% in 6 h, while the uncoated NLC attained 96.02% after 7 h. These results highlight that decoration with hyaluronic acid not only accelerates the release of polyphenols but also sustains the overall effectiveness of the system, suggesting enhanced potential for the controlled delivery of bioactive compounds.

The Korsmeyer–Peppas model provided the best regression coefficient for all NLC formulations, decorated and undecorated with hyaluronic acid. The exponent “*n*” indicated the mechanism of polyphenol release, with values ranging from 0.34 to 0.67 (Table 2). In the case of NLC-*ArnicaM, n* = 0.33 indicates a Fickian diffusion mechanism, while for the other formulations (0.43 < *n* < 0.89), the release follows a non-Fickian behaviour, indicating that polyphenols were released by an anomalous transport, based on a combination of diffusion and the lipid matrix degradation that controls their release from the NLCs [65,66]. The existence of hyaluronic acids on the surface of the NLCs results in the creation of an extra layer surrounding them, which contributes to the controlled release of polyphenols [15].

### 3.6. In Vitro Assignment of Cell Viability and Cytotoxic Potential, by MTS and LDH

**Figure 7:**
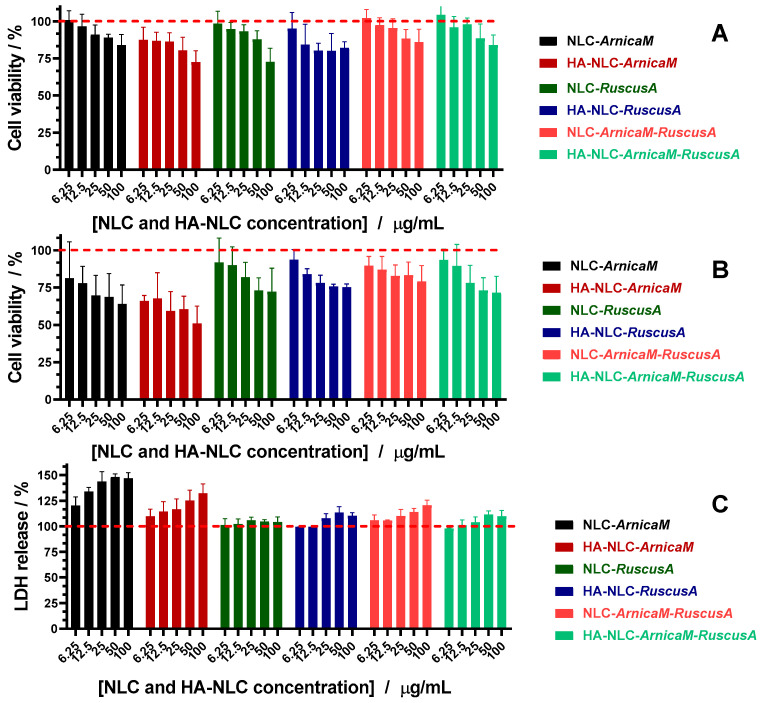
In vitro assignment of cell viability and cytotoxic potential, by MTS. (**A**) After 24 h; (**B**) after 48 h and LDH test (**C**).

The biocompatibility of NLCs and HA-NLCs coopting the active herbals was investigated on BJ fibroblast cells at 24 and 48 h after treatment with concentrations of 0–100 µg/mL (Figure 7). With increasing concentration, a moderate decrease in cell viability was detected, which does not reveal a potential cytotoxic risk, except for HA-NLC-*ArnicaM* at 48 h which shows a more advanced trend decrease in the cell viability. Despite an intrinsic cytotoxicity of the *ArnicaM* extract, the cell viability values confirm that NLCs and HA-NLCs can be considered safe at all tested concentrations, with minor exceptions. The treatment with 25 and 50 µg/mL NLCs and HA-NLCs resulted in an appropriate viability > 80%, especially for NLCs with dual content of herbal extracts.

In the case of NLC-*ArnicaM,* the presence of higher concentrations of terpene lactones may most likely be responsible for triggering apoptosis [67]. However, the combination of *ArnicaM* with *RuscusA* in NLCs exploits pharmacological synergy with the achievement of a superior biological effect. The likelihood of *RuscusA* to potentiate the venotonic and anti-inflammatory effect allows *ArnicaM* to remain below the cytotoxicity threshold [68]. *RuscusA* may also act as a metabolic “shielding” agent, reducing the oxidative stress that *ArnicaM* could induce at the mitochondrial level. The attenuation of cell lysis and the protection of the BJ fibroblast cell membrane can also be considered a critical point. The coaptation of the two extracts in NLCs avoids the interaction of some aggressive phytocompounds, e.g., sesquiterpene lactones, with the phospholipids of the BJ cell membrane. A notable aspect is represented by the behaviour of HA-decorated NLCs, where a more profound decrease in cell viability was detected at concentrations of 50 and 100 μg/mL. The hyaluronic acid coating may have favoured endocytosis, driving the phytocompounds directly into endosomal compartments. This behaviour may result in the local accumulation of potentially aggressive concentrations of phytochemicals at the BJ cells’ membrane surface, increasing the risk of membrane disruption (cell lysis).

To determine whether the cytotoxicity induced by NLC-*ArnicaM* is caused by cell lysis, LDH release was monitored after a 48 h treatment (Figure 7C). LDH enzyme activity is a reliable indicator of cell membrane integrity. NLC- and HA-NLC-*ArnicaM* led to a prominent increase in LDH levels (Figure 7C). When a cell is damaged by membrane destruction, LDH “leaks” from the cytoplasm to the outside, increasing the percentage of LDH. The results obtained on the cell injury marker—LDH—confirm that one of the main mechanisms is related to membrane lysis. Our NLC results are in correlation with previous studies reported for *ArnicaM* extract, which induced an increase in LDH levels for concentrations higher than 100 µg/mL [69]. For other NLCs, LDH levels did not undergo relevant changes, indicating that membrane lysis is not the main mechanism by which cell viability is affected.

### 3.7. Wound Healing Potential of NLCs and HA-Decorated NLC-ArnicaM-RuscusA

**Figure 8:**
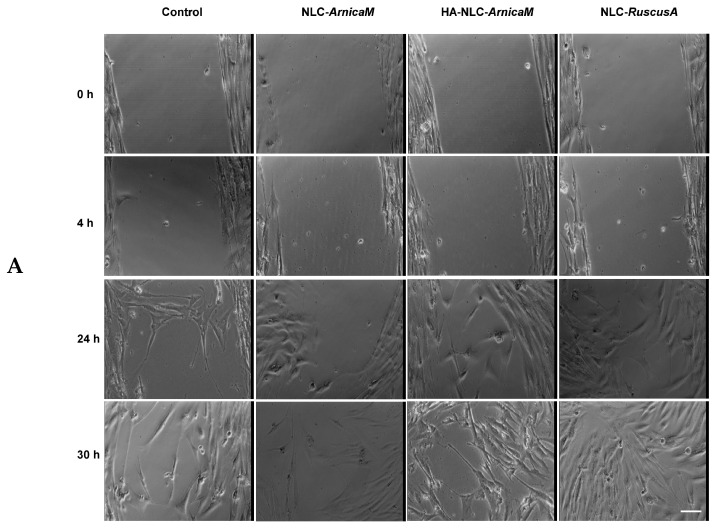

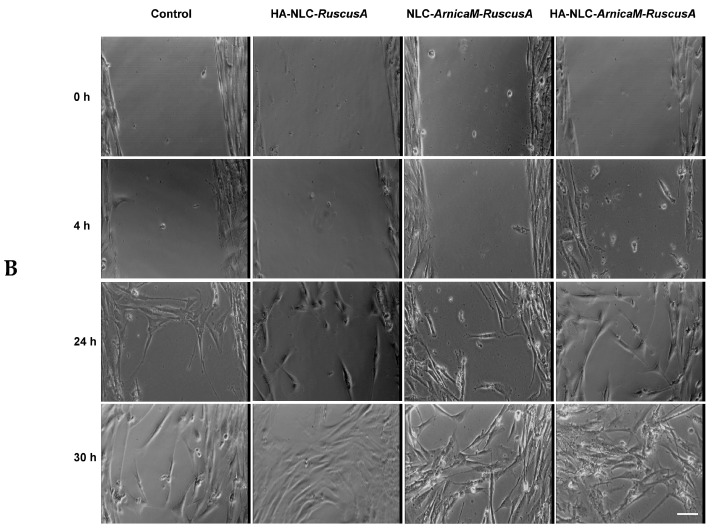
Representative bright field images, with visualisation of wound closure, in the presence of various types of NLC- and HA-NLC-herbal extracts (**A**,**B**). The scale bar is 20 µm and is the same for all images.

The ability of NLC- and HA-NLC-plant extracts to promote wound healing was assessed using the scratch test. At the initial moment of the scratch, a large wound, delimited by clear edges, was obtained (Figure 8). Representative bright-field images are presented in Figure 8A,B for several time points up to 30 h, when the wound was almost completely closed for the two categories of nanocarrier systems loaded with plant extracts. Thus, after 4 h of treatment with NLC- and HA-NLC-plant extracts, it was observed that all conditions looked approximately similar, with almost no migration in the wound area. In contrast, 24 h of treatment led to the modification of the cellular morphology (fusiform aspect), with massive migration in the wound area. An extension up to 30 h shows that the wound was almost closed for all investigated NLCs. In parallel, for the control conditions, the cells migrate, but there are visible spaces between the cells. Cells treated with hybrid nanocarriers, namely HA-decorated NLCs, better close the wound caused by the wound, the cells reaching confluence when treated with HA-NLC-*RuscusA,* followed by HA-NLC-*ArnicaM-RuscusA*. The ability of these hybrid lipid nanocarriers to accelerate the closure of wounds on BJ fibroblast cells is explained by the synergy between the structure and abilities of the nanocarrier, correlated with the active phytocompounds delivered and released in a controlled manner: *(i). Hyaluronic acid used for the decoration* of *the NLC* is a recognition-specific element by the CD44 receptors expressed on the surface of BJ fibroblasts [8]. HA acts as a key, binding to the CD44 receptors, thus facilitating the rapid internalisation of *ArnicaM* and *RuscusA,* most likely by endocytosis. Furthermore, the interaction between HA and the CD44 receptor can trigger intracellular signalling cascades [70] that stimulate cell motility, causing fibroblasts to migrate more rapidly towards the free area, promoting wound closure. *(ii). Synergy of ArnicaM and RuscusA extracts.* For instance, *ArnicaM* contains sesquiterpene lactones that, in controlled doses, modulate the inflammatory response and stimulate cellular metabolism [71]. This provides the metabolic fuel necessary for fibroblasts to divide and migrate. *RuscusA,* rich in steroidal saponins, improves cellular resistance and contributes to the stability of the BJ cell membrane [29]. *(iii). The dynamics of release* also play a defining role in the scratch assay, as healing is the result of two processes, cell migration and proliferation. The gradual release of the phytochemicals reduces the oxidative stress caused by the mechanical “wound” on BJ fibroblasts. Due to the slow release, BJ fibroblast cells receive a constant stimulus for migration, so that the scratch closes significantly faster compared to untreated cells. All these considerations confirm the hypothesis of a remarkable effectiveness of HA-NLCs, and especially HA-NLC-*RuscusA*, in promoting wound healing.

**Figure 9:**
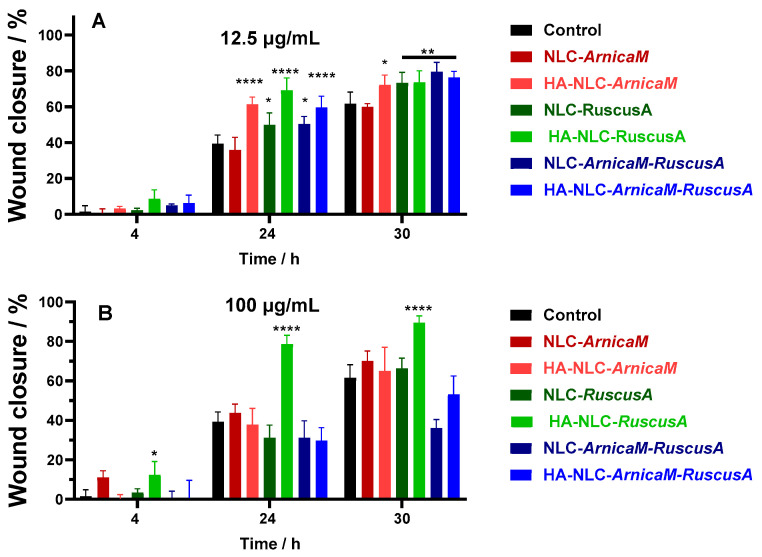
Scratch-wound closure monitored over time in BJ cells, untreated or treated with 12.5 µg/mL (**A**) and 100 µg/mL (**B**) of NLCs and HA-NLCs entrapping herbal extract. Data are expressed as mean ± SD, *n* = 3. *p* values were calculated using ANOVA analysis with Tukey’s multiple comparison post-test. * *p* < 0.05, ** *p* < 0.01, **** *p* < 0.0001.

Quantitative analysis was done for all conditions tested and the percentage of the wound closure is presented for 12.5 µg/mL and 100 µg/mL concentrations (Figure 9). Comparative analysis of cells treated with 12.5 µg/mL of NLCs and HA-NLCs shows that the first biological difference is observed at 24 h, where three distinct groups can be observed (Figure 9A): *(i)* NLCs with weak effect compared to the control (NLC-*ArnicaM*), *(ii)* moderate wound closure effect (NLC-*RuscusA* and NLC-*ArnicaM-RuscusA*), and *(iii)* strong wound closure action (case of all HA-NLCs). At 30 h, the wound closure process is almost complete, with the observation that at this time, there is almost no difference between NLCs and HA-NLC-plant extracts, except for NLC-*ArnicaM*, which did not show a healing effect when applied. According to the results obtained, HA-NLCs promote a faster healing response after application, but this slows down after 24 h, while NLCs show continued efficacy, reaching a similar result at 30 h. These results confirm the adaptability of HA-NLCs as effective candidates for pharmacological application, with the possibility of reapplication after 24 h to help maintain faster cell migration.

*RuscusA* has anti-inflammatory and antioxidant action, as well as effects on microcirculation [28], but no wound healing studies have been reported for this extract or on nanoformulations of it. This is the first study to show an increase in cell migration, which is beneficial for wound healing. On the other hand, *ArnicaM* is used as an anti-inflammatory and antimicrobial agent, and in the treatment of various skin lesions [23,29,72]. It has been previously shown that *ArnicaM* can enhance the in vitro migration of fibroblasts [29,70].

When cells were exposed to 100 µg/mL, a different result was determined compared to the lower concentration (Figure 9B). The most effective treatment was HA-NLC-*RuscusA*, while for the other treatments, the results were largely similar to the control conditions. This phenomenon can be interpreted based on several mechanistic perspectives. A first aspect would be reaching a “therapeutic window” at 12 µg/mL, while a higher dose may lead to an inhibition or slowing of the wound closure of BJ fibroblasts. Although NLCs are biocompatible at 100 µg/mL, the density of particles in the culture medium is extremely high, which may disrupt the integrity of the plasma fibroblasts’ membrane. As such, instead of stimulating migration, the dose of 100 µg/mL may induce metabolic stress or even cellular micro-injuries [73], forcing cells to enter internal repair processes, instead of dividing and migrating to close the “scratch”. Also, the concentration of 100 µg/mL modifies the rheological properties/viscosity of the culture medium, and implicitly, a diffusion restriction occurs. Cell migration is an active process that requires cytoskeletal rearrangement. In a much more viscous and denser environment, the hydrodynamic resistance opposing cell movement is greater, resulting in a much lower wound closure rate [74]. Also, the efficiency of the scratch-wound assay depends on the ability of cells to detect free space and migrate towards it. At 100 μg/mL, the cell surface and extracellular space (hydrodynamic volume) are practically “saturated” with NLCs. This physical agglomeration may create a steric barrier (for example, BJ fibroblasts may have blocked surface CD44 receptors), which may inhibit the signalling required for locomotion. Finally, *ArnicaM* and *RuscusA* extracts are rich in bioactive compounds (sesquiterpene-lactones, flavonoids, saponins) that may exhibit a pro-oxidant effect at high doses [75]. At 12 μg/mL, NLCs and HA-NLC-*ArnicaM* and/or *RuscusA* can stimulate various signalling pathways that promote proliferation, while at 100 μg/mL, intracellular accumulation of NLCs can generate high levels of reactive oxygen species (ROSs). This high oxidative stress leads to cell cycle arrest, thus halting the proliferation necessary for wound healing.

### 3.8. Cell Morphology and Cytoskeleton Organisation

**Figure 10:**
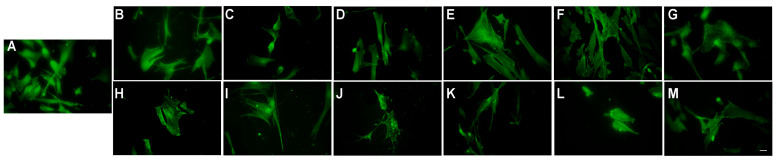
BJ cells’ morphological changes evidenced by fluorescence microscopy: control (**A**), NLC-*ArnicaM* ((**B**) 12.5 µg/mL; (**D**) 100 µg/mL), HA-NLC-*ArnicaM* ((**C**) 12.5 µg/mL; (**E**) 100 µg/mL), 100 µg/ mL NLC-*RuscusA* ((**F**) 12.5 µg/mL; (**H**) 100 µg/mL), HA-NLC-*RuscusA* ((**G**) 12.5 µg/mL; (**I**) 100 µg/mL), NLC-*ArnicaM-RuscusA* ((**J**) 12.5 µg/mL; (**L**) 100 µg/mL), HA-NLC-*ArnicaM-RuscusA* ((**K**) 12.5 µg/mL; (**M**) 100 µg/mL). Magnification of all images is 40×. The scale bar is 10 µm and is the same for all images.

Cell morphological changes induced by the NLC- and HA-NLC-plant extracts were viewed for 12.5 and 100 µg/mL by analysing the actin filaments. As expected, BJ fibroblast cells have an elongated and spindle-shaped cell body, specific to fibroblast cells’ morphology (Figure 10A). Because of the long and well-organised actin filaments, which are highly polarised along the main axis, cells also tend to grow more aligned together. Compared to control cells, when treated with 12.5 mg/mL NLC- and HA-NLC-plant extracts, the cells maintained their elongated look, exhibiting a spindle-shaped morphology, with actin fibres aligned along the axis (Figure 10). With increasing concentration, the cells became smaller, exhibiting a reduction of the actin filaments.

**Figure 11:**
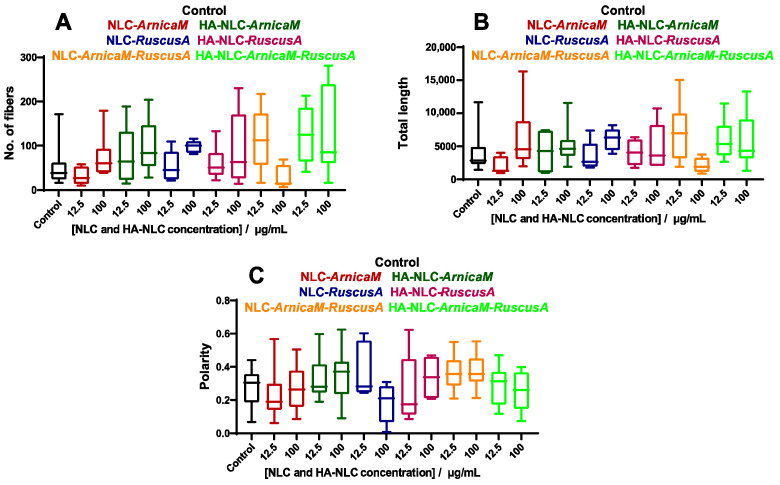
NLC- and HA-NLC-herbal extracts remodel the cytoskeleton of BJ cells. (**A**) Number of fibres, (**B**) total length of fibres, and (**C**) the fibres’ polarity, as determined from fluorescence images using the fibre score algorithm.

The images were further analysed using a MATLAB code modified as described previously [76], and the number of cytoskeleton fibres, total length, and polarity were calculated and are reported in Figure 11. Upon treatment with NLC-*ArnicaM,* the parameters exhibit only a small change, indicating that the cytoskeleton has maintained its integrity independent of the concentrations applied. However, when HA was added, all parameters showed slightly increased values, which can be determined by the stimulation of cell migration. For NLC-*RuscusA,* the values were similar compared to the control; however, a slight increase in the parameters was observed for HA-NLC-*RuscusA*, which indicates a promotion of cell migration.

NLC-*ArnicaM-RuscusA* induces a strong actin fragmentation when cells were treated with 100 µg/mL, although the polarisation was not affected. For HA-decorated NLC-*ArnicaM-RuscusA*, an increase in the number of fibres and their length was observed, with no changes to their polarity independent of the concentration used. This can be correlated with the increase in fibroblast cell mobility.

## 4. Conclusions

The structural design as well as antioxidant and healing effect of conventional NLCs and HA-modified NLCs carrying a complex phytochemical profile of *ArnicaM* and *RuscusA* extracts have been investigated. These nanocarriers exhibited main diameters ranging from 145 nm to 180 nm, electrokinetic potential between −45 mV and −62 mV, and an ideal hosting ability for both plant extracts, with encapsulation efficiency exceeding 96%. The phytochemicals’ release profiles from NLCs and HA-modified NLCs revealed a distinct profile, influenced by the HA and the category of phytochemical extract entrapped. Decoration of NLCs with HA led to a notable increase in the rate of polyphenol release, e.g., HA-NLCs reached a percentage release of *ArnicaM* of 96.7% after 6 h versus 90.02% of NLC after 7 h. A similar trend was observed for the dual NLCs with mixed content of *ArnicaM* and *RuscusA.*

The antioxidant superiority of NLCs and HA-NLC-*ArnicaM* over NLCs with *RuscusA* or dual ones has been shown. *ArnicaM* wins the antioxidant competition while *RuscusA* proved excellent for accelerating the wound closure process in BJ fibroblast cells. NLCs and HA-NLCs entrapping *ArnicaM* exhibited the highest capacity to neutralise DPPH free radicals, e.g., 79.38 ± 0.25%, 74.69 ± 0.5%. The activity of NLC-*ArnicaM*-*RuscusA* was more moderate in the direct inhibition of free radicals. The complex phytomolecules in NLC-*ArnicaM-RuscusA* may limit the transfer of electrons to DPPH radicals, which translates into a lower reaction rate in the neutralisation of radicals. Decoration of NLCs with HA slowly decreased the antioxidant values as compared to NLC-plant extracts without HA. Through HA anchoring, a sequestration of phytochemical antioxidants has been produced, and the active groups of antioxidants become locked in the polymer network, thus reducing the number of active centres available for neutralizing DPPH radicals.

The biocompatibility of HA-NLCs coopting the active herbals shows that *ArnicaM* together with *RuscusA* exploits the emergence of a pharmacological synergy. HA-NLC-*ArnicaM*-*RuscusA* functioned as a buffer between the potentially toxic phytocompounds of *ArnicaM* and fibroblast cells, while *RuscusA* allowed the achievement of biological efficacy without compromising the integrity of the cell membrane. Although lipid nanocarriers entrapping only *ArnicaM* led to a prominent increase in the cellular LDH marker, concentrations of 25 and 50 µg/mL resulted in the maintenance of a viability > 80%, especially for NLCs with dual vegetable extract content. The co-presence of *RuscusA* with *ArnicaM* may act as a metabolic “shielding” agent, reducing oxidative stress and/or mitigating the risk of cell lysis that certain aggressive phytochemicals in *ArnicaM* could induce.

The hybrid NLCs demonstrated remarkable efficacy in accelerating the closure of scratch wounds induced on human skin-derived BJ fibroblast cells. The quantitative wound closure analysis for 12.5 µg/mL and 100 µg/mL highlighted clear biological differences observed at 24 h, which fall into three distinct groups: (*1*) NLCs with weak effect compared to the control (for NLC-*ArnicaM*), (*2*) moderate wound healing effect (for NLC-*RuscusA* and NLC-*ArnicaM-RuscusA*), and (*3*) strong wound healing effect (case of all HA-NLC-phytochemicals). Several aspects that sustain the hypothesis of a remarkable efficiency of HA-NLC-*ArnicaM-RuscusA* to promote wound healing can be closely correlated with the following: *i.* the interaction between HA and CD44 receptors, which facilitates the internalisation of *ArnicaM* and *RuscusA* and can also trigger intracellular signalling cascades that stimulate cell motility and a faster fibroblasts migration. *ii.* The active phytocompounds in *ArnicaM* and *RuscusA* can stimulate cellular metabolism, acting as metabolic fuels necessary for fibroblasts to divide and migrate. *iii.* Due to the gradual release dynamics, the oxidative stress caused by mechanical wounding on fibroblasts is reduced, and BJ cells receive a constant stimulus for migration and proliferation. The BJ cells’ morphological changes have been evidenced by fluorescence microscopy. NLC-*ArnicaM* induced a small change in the parameters, indicating that the cytoskeleton maintained its integrity regardless of the applied concentrations. In contrast, for NLCs modified with HA, all parameters showed slightly increased values, which may be associated with the stimulation of cell migration. For HA-decorated NLC-*ArnicaM-RuscusA*, fibres proliferation was observed, without changes in their polarity, a behaviour that can be correlated with increased cellular mobility.

The results of this work facilitate the design of hybrid lipid nanocarriers for mixed plant extracts with improved stability and wound healing effect, thus contributing to the future development of functional topical nanostructured formulations that can stimulate signalling pathways and promote an appropriate cell regeneration necessary for wound healing, simultaneously with an intracellular accumulation of nanocarriers that maintains a low level of reactive oxidizing species.

## Figures and Tables

**Table 1:** The IC_50_ values of DPPH radical scavenging activity.

NLC Formulations	IC_50_ [mg/mL]
NLC-*ArnicaM*	0.222 ± 0.010
HA-NLC-*ArnicaM*	0.548 ± 0.004
NLC-*RuscusA*	0.723 ± 0.019
HA-NLC-*RuscusA*	0.801 ± 0.048
NLC-*ArnicaM*-*RuscusA*	0.676 ± 0.034
HA-NLC-*Arnica*M-*RuscusA*	0.659 ± 0.014

**Table 2:** Kinetic models parameters obtained for the phytochemicals release.

NLC and HA-NLC Formulations	Order 0		Order 1		Higuchi		Hixson–Crowell		Peppas–Korsmeyer		
	$\%R=k_{0}t$		$\ln\left(100-\%R\right)=k_{1}t$		$\%R=k_{2}\surd t$		$\sqrt[{3}]{100-\%R}=k_{3}t$		$\%R=k_{4}\sqrt[{n}]{t}$		
	*R* ^2^	*k*_0_	*R* ^2^	*k*_1_	*R* ^2^	*k*_2_	*R* ^2^	*k*_3_	*R* ^2^	*k*_4_	*n*
NLC-*ArnicaM*	0.9603	11.949	0.890	0.195	0.9141	39.388	0.7543	0.041	0.9974	1.553	0.34
HA-NLC-*ArnicaM*	0.9985	16.376	0.8384	0.506	0.9882	56.616	0.7487	0.083	0.9987	2.781	0.49
NLC-*ArnicaM-RuscusA*	0.9696	12.936	0.8899	0.283	0.9044	38.209	0.8699	0.038	0.9757	2.593	0.52
HA-NLC-*ArnicaM-RuscusA*	0.9800	15.905	0.8299	0.594	0.9934	49.967	0.9067	0.056	0.9958	3.267	0.67

## Data Availability

The raw data supporting the conclusions of this article will be made available by the authors on request.

## Abbreviations

HA	hyaluronic acid
NLC	Nanostructured Lipid Carriers
SLN	Solid Lipid Nanoparticles
*ArnicaM*	*Arnica montana*
*RuscusA*	*Ruscus aculeatus*
DLS	dynamic light scattering
Z_ave_	mean particle diameter
PdI	polydispersity index
GAE	gallic acid equivalents
DSC	differential scanning calorimetry
DPPH	2,2-diphenyl-1-picrylhydrazyl
BJ	human skin fibroblast cells
LDH	lactate dehydrogenase
